# A computational method to estimate the relative biological effectiveness and tumor control probability for low-LET proton irradiations

**DOI:** 10.1371/journal.pone.0341352

**Published:** 2026-03-20

**Authors:** Chun-Chieh Chan, Kuang-Lung Hsueh, Chung-Yu Lai, Ya-Yun Hsiao

**Affiliations:** 1 Department of Computer Science and Information Engineering, National Formosa University, Yunlin, Taiwan; 2 Protein Science and Biophysics Department, WuXi Biology, WuXi AppTec, Shanghai, China; 3 Department of Medical Equipment Development and Application, Hungkuang University, Taichung, Taiwan; 4 Department of Medical Imaging and Radiological Sciences, Chung Shan Medical University, Taichung, Taiwan; 5 Department of Medical Imaging, Chung Shan Medical University Hospital, Taichung, Taiwan; Siksha O Anusandhan University Institute of Technical Education and Research, INDIA

## Abstract

A constant relative biological effectiveness (RBE) value of 1.1 is used for proton therapy (PT) in many clinical treatment plans. However, several studies show that RBE varies with proton energy, linear energy transfer (LET), and oxygen concentration. This study presents a computational method based on the linear quadratic (LQ) and repair-misrepair-fixation (RMF) models to calculate tumor control probability (TCP) under varying oxygen conditions. We analyze the impact of hypoxia on the parameters of the LQ model, focusing on the ratio and RBE. The proposed method allows for TCP calculations across different oxygen concentrations and for various ion therapies, such as proton and carbon ion therapy. Our results show that increasing the LET from 1 to 12 keV/μm enhances TCP from 61% to 98% under aerobic conditions (21% O_2_), 45% to 98% under moderately hypoxic conditions (2% O_2_), and from 1% to 48% under severely hypoxic conditions (0.1% O_2_). These findings are compared with clinical trial data, demonstrating that hypoxia significantly affects TCP for low-LET radiations.

## Introduction

The number of centers and patients that use proton therapy (PT) is increasing, and as of June 2024, 123 centers worldwide are in clinical operation and close to 320,000 patients have received PT [1]. PT offers a more conformal dose distribution than traditional X-ray therapy (XT), which is due to the fact that proton beams deposit their energy in regions with minimized scattering in the surrounding healthy tissues [2]. The main feature of a proton beam is the Bragg peak, which is in a well-defined and highly localized region at the end of the proton beam [3].

The dose recommendation for PT depends on the relative biological effectiveness (RBE), which is the ratio of the doses for photons relative to protons to reach the same biological endpoint [4–7]. Protons are also considered to be a low linear energy transfer (LET) radiation with energy dependence and the peak and the spread-out of Bragg peak regions contain rare high LET-like radiation tracks [8,9]. Although the LET is higher within the peaks, the spreading effect leads to an overall increase in the LET value. A constant RBE value of 1.1 is used for PT in many clinical treatment plans but studies show that the value for RBE varies with cell type, radiosensitivity and LET [10,11].

To calculate the variable RBE values, radiation doses can be calculated using the linear quadric (LQ) model [12] which involves radiosensitivity and curve-fitting parameters α and β. Parameter α has been shown to be dependent on LET [13–15] and oxygen concentration [16]. To explicitly account for the impact of oxygen on radiosensitivity within the LQ framework, the effects of oxygen on cells can be quantified in terms of the oxygen enhancement ratio (OER), the ratio of hypoxic dose to aerated dose needed to achieve the same biological outcomes [17]. Alternatively, OER may also be defined as the ratio of biological effects such as DNA double strand break (DSB) yields or cell killing at the same dose [18]. The values for α and β in the LQ model can be determined using *in vitro*/*in vivo* cell survival experiments or mathematical models [19–24], such as the microdosimetric-kinetic model [20], LQ-based phenomenological RBE models (e.g., Wedenberg model [14], Carabe-Fernandez model [13,21], McNamara model [22]), and a mechanistic DSB-based repair-misrepair-fixation (RMF) model [25]. Variations in α and β arising from differences in LET and oxygenation conditions, as quantified by the OER, lead to corresponding changes in the predicted RBE.

The photon iso-effective or equivalent dose (EQD) [26] and tumor control probability (TCP) [27] are used to determine the RBE effects in the outcome of PT treatment [26]. The EQD is the photon dose that is required to achieve the same biological outcome as that for cells that are irradiated by proton beams. TCP is the probability that a tumor is eradiated or controlled by a specific dose and is essential for comparing conventional radiotherapy with advanced modalities (e.g., proton vs. carbon ion therapy) to determine the best approach for specific cancer types. TCP contributes to the evaluation of novel combination therapies, such as ion therapy with radiosensitizers targeting tumor regions [28].

TCP depends on the fractionated dose [29], LET [8] and the biological effect of cells, such as repopulation [30], repair [31] and reoxygenation [32]. Repair is affected by oxygen concentration because the oxygen fixation hypothesis states that the presence of oxygen fixes the DNA damage and the damage cannot be repaired [33]. That is, under normal conditions, cells have mechanisms to repair radiation-induced DNA damage, especially when the damage is reversible. However, the presence of oxygen can “fix” or stabilize the DNA damage, making it permanent. Oxygen reacts with the DNA radicals (the intermediates created during the initial damage), converting them into more chemically stable, but unrepairable forms. This prevents the repair mechanisms from reversing the damage. Furthermore, the degree of tumor hypoxia is a plausible mechanism for TCP reduction [34]. Given that cellular-level hypoxia can fluctuate over time [35], insufficient reoxygenation time between fractions may result in a reduced TCP [36].

This study determines the effect of hypoxia on the treatment outcome in terms of LQ-model derived RBE and TCP. A dose-modifying factor (DMF) [26] and the RMF model [25] are used to calculate the values of the parameters α and β for the LQ model. The RBE for PT relative to XT and the probable gain in tumor control for PT over XT is also determined. Some approaches to determine TCP under hypoxic conditions requires patient data and/or functional hypoxia imaging modality [37–39]. Other methods offer theoretical models but computationally expensively [40–42]. We proposed a simple and fast LQ model-based algorithm to calculate TCP, which is in the analytic form and can be applied to different ion treatments. Our results show good agreement with experimental/clinical evaluations and theoretical predictions. The TCP values are compared with those for clinical trial data to determine the effect of hypoxia on the outcomes of treatment plans.

## Methods

### Cell data and clinical data

For this study, we utilized published data from several cell lines which were drawn from *in vitro* experiments reported by [15,43–46], and prostate cancer patient data (in Tables 4 and 5) which were obtained from clinical trials [47,48]. A total of 393 patients with T1b to T2b stage prostate cancer and prostate-specific antigen levels below 15 ng/mL were randomized between January 1996 and December 1999 and received treatment at two academic institutions in the United States [47,48]. The analysis was structured by first calculating the α and β parameters under different oxygen concentrations, followed by the application of our algorithm to calculate TCP for a range of LET values.

**Table 1:** Predictions for EQD and TCP for proton beams with various LET values under aerobic conditions (21% O_2_).

LET (keV/μm)	$\alpha_{p}$	$\beta_{p}$	EQD_RMF_ (Gy)	TCP (%)	EQD_DMF_ (Gy)	TCP (%)	EQD_clinical_ (Gy)	TCP(%)
1	0.344 ± 0.069	0.069 ± 0.0004	66 ± 13	61 ± 20	65 ± 15	61 ± 23	74 ± 9	71 ± 11
4	0.376 ± 0.071	0.080 ± 0.0003	73 ± 14	71 ± 17	70 ± 18	67 ± 24	74 ± 9	71 ± 11
7	0.431 ± 0.074	0.089 ± 0.0003	83 ± 16	81 ± 13	78 ± 21	76 ± 21	74 ± 9	71 ± 11
12	0.820 ± 0.090	0.112 ± 0.0005	144 ± 24	98 ± 2	132 ± 34	97 ± 4	74 ± 9	71 ± 11
18	1.114 ± 0.096	0.124 ± 0.0006	188 ± 30	99 ± 1	174 ± 43	99 ± 1	74 ± 9	71 ± 11
23	1.381 ± 0.098	0.151 ± 0.0007	233 ± 36	100 ± 0	211 ± 52	100 ± 1	74 ± 9	71 ± 11

**Table 2:** Predictions for EQD and TCP for proton beams with various LET values under moderately hypoxic conditions (2% O_2_).

LET (keV/μm)	$\alpha_{p}$	$\beta_{p}$	EQD_RMF_ (Gy)	TCP (%)	EQD_DMF_ (Gy)	TCP (%)	EQD_clinical_ (Gy)	TCP(%)
1	0.315 ± 0.063	0.049 ± 0.00020	67 ± 15	45 ± 23	66 ± 15	44 ± 23	74 ± 8	55 ± 12
4	0.379 ± 0.065	0.057 ± 0.00023	80 ± 16	63 ± 20	77 ± 17	59 ± 22	74 ± 8	55 ± 12
7	0.463 ± 0.069	0.063 ± 0.00182	95 ± 19	78 ± 14	91 ± 19	74 ± 17	74 ± 8	55 ± 12
12	0.905 ± 0.083	0.080 ± 0.00042	173 ± 30	98 ± 2	164 ± 33	97 ± 3	74 ± 8	55 ± 12
18	1.213 ± 0.088	0.089 ± 0.00049	227 ± 38	99 ± 1	215 ± 43	99 ± 1	74 ± 8	55 ± 12
23	1.610 ± 0.091	0.109 ± 0.00042	299 ± 48	100 ± 0	280 ± 56	100 ± 0	74 ± 8	55 ± 12

**Table 3:** Predictions for EQD and TCP for proton beams with various LET values under extremely hypoxic conditions (0.1% O_2_).

LET (keV/μm)	$\alpha_{p}$	$\beta_{p}$	EQD_RMF_ (Gy)	TCP (%)	EQD_DMF_ (Gy)	TCP (%)	EQD_clinical_	TCP(%)
1	0.118 ± 0.045	0.007 ± 0.00004	64 ± 30	1 ± 3	64 ± 30	1 ± 3	74 ± 6	3 ± 2
4	0.144 ± 0.047	0.008 ± 0.00005	77 ± 34	3 ± 6	76 ± 34	3 ± 6	74 ± 6	3 ± 2
7	0.170 ± 0.048	0.009 ± 0.00005	91 ± 37	6 ± 10	89 ± 38	5 ± 10	74 ± 6	3 ± 2
12	0.334 ± 0.059	0.012 ± 0.00008	173 ± 63	48 ± 38	169 ± 65	45 ± 40	74 ± 6	3 ± 2
18	0.465 ± 0.064	0.013 ± 0.00010	239 ± 83	78 ± 26	234 ± 87	76 ± 29	74 ± 6	3 ± 2
23	0.588 ± 0.065	0.017 ± 0.00011	302 ± 102	90 ± 13	293 ± 108	89 ± 15	74 ± 6	3 ± 2

**Table 4:** Comparison of biochemical tumor control probability (bTCP) for patients who received proton treatments (LET ≈ 1 keV/μm) for prostate cancer with 11 proton fractions.

Data		dose per fraction (Gy)	No of fractions	observed bTCP (%)	95% confidence interval	oxygen conc. (%)	predicted bTCP_RMF_ (%)	predicted bTCP_DMF_ (%)	predicted bTCP_clinical_ (%)
[47]	photon	1.8	28	61.4	54.6-68.3	21	65 ± 7	65 ± 7	67 ± 5
	proton	1.64	11						
						2	47 ± 7	48 ± 7	50 ± 5
						0.1	2 ± 2	2 ± 1	2 ± 1

**Table 5:** Comparison of biochemical tumor control probability (bTCP) for patients who received proton treatments (LET ≈ 1 keV/μm) for prostate cancer with 16 proton fractions.

Data		dose per fraction (Gy)	No of fractions	observed bTCP (%)	95% confidence interval	oxygen conc. (%)	predicted bTCP_RMF_ (%)	predicted bTCP_DMF_ (%)	predicted bTCP_clinical_ (%)
[47]	photon	1.8	28	80.4	74.7-86.1	21	74 ± 7	74 ± 7	77 ± 5
	proton	1.64	16						
						2	59 ± 9	59 ± 9	62 ± 6
						0.1	3 ± 2	3 ± 2	3 ± 2

### Method development

This study introduces a TCP calculation method that builds upon the LQ and RMF models. While the LQ model traditionally estimates cell survival fractions based on the radiosensitivity parameters α and β, the effects of hypoxia on RBE is not accounted. We have combined their model with RMF model [25]. This combination provides the RBE equation to account for hypoxic conditions, thereby enabling more accurate predictions of RBE. Meanwhile, TCP is an essential metric for predicting treatment success. To compare different radiation dose schedules and accurately evaluate TCP, calculating EQD is necessary, as it incorporates the radiosensitivity parameters α and β. We assume uniform hypoxia which allows us to isolate the effects of α and RBE as a function of LET and oxygen concentration without introducing geometry-dependent confounding factors. It follows many widely cited TCP studies that investigate the hypoxia effect analytically [36,40,42]. We will present the calculations for RBE, OER, EQD, and TCP separately.

#### RBE calculations.

The RBE is defined as the ratio of the dose of low-LET reference radiation, such as X-rays, to the fractionation dose *d* of any other radiation that is required to achieve an equal biological effect [12] and is defined as:


$$RBE=\frac{d_{x}}{d_{p}}$$ (1)

where subscripts *P* and *X respectively* denote protons and X-rays.

The survival fraction (SF) is defined using the LQ equation as:


$$SF=e^{-\left(\alpha D+\beta D^{\text{2}}\right)}$$ (2)

where SF denotes the survival fraction of cells for dose *D,* using the curve fitting parameters *α* and *β*. Fractionation effects are expressed as the survival rate after *n* photon fractions at dose *d* with the curve-fitting parameters *α* and *β* for photons and is written as:


$$SF=e^{\left[n\left(-\alpha d-\beta d^{2}\right)\right]}$$ (3)

Eq. (3) can be rewritten as for X-rays:


$$Effect=n\left(\alpha d+\beta d^{2}\right)$$ (4)

and for protons:


$$Effect_{p}=n_{p}\left(\alpha_{p}d_{p}+\beta_{p}{d_{p}}^{2}\right)$$ (5)

where *α* and *β*, $\alpha_{p}$, and $\beta_{p}$ are the *α* and *β* parameters that are respectively defined in Eq. (2) for X-rays and proton exposure. *d* and $d_{p}$ are the X-ray and proton delivery doses, respectively.

The RBE can be written as [15]:


$$RBE=\frac{\alpha }{\beta }\frac{\left(-1+\sqrt{1+\frac{4\left(\alpha_{p}d_{p}+\beta_{p}d_{p}^{2}\right)}{\frac{\alpha^{2}}{\beta }}}\right)}{2d_{p}}$$ (6)

In RMF model [25], assume that complex DSBs comprising *j* or more lesions—such as strand breaks, damaged bases, or abasic sites—are intrinsically irreparable. The presence of multiple lesions within a single DSB is enough to permanently prevent enzymatic repair or the proper ligation of the break ends. Then $f_{R}$ is the fraction of initial DSBs that can be re-joined and is defined as:


$$f_{R}=\frac{\text{1}}{\sum }\sum_{i=2}^{j-1}\sum_{i}$$ (7)

the LQ-based cell survival fraction curve fitting parameters α and β are expressed as:


$$\alpha =\left[1-f_{R}\left(1-\theta \right)\right]\sum +\kappa {\bar{z}}_{F}{\left(f_{R}\sum \right)}^{2}$$ (8)

$$\beta =\left(\kappa /2\right){\left(f_{R}\sum \right)}^{2}$$ (9)

The parameter *j* refers to the minimum number of damaged sites within a DSB that prevents the DNA from being properly repaired. The summation in Eq. (12) from *j* = 2 to *j* – 1 because every DSBs consists of at least two strand breaks. ∑ is the total number of DSB per cell per Gy and $\Sigma_{i}$ is the expected number of DSBs per cell per Gy that consist of exactly *i* lesions. The DSB yields were estimated using Monte Carlo Damage Simulation (MCDS) software [49] and are provided in S1 File. θ is defined as the fraction of DSBs that undergo lethal first-order mis-repair and damage fixation and κ is defined as the fraction of initial DSBs that undergo pairwise damage interactions. ${\bar{z}}_{F}$ is the frequency mean specific energy for a spherical target that is composed of water with diameter *d* and is defined as follows.


$${\bar{Z}}_{F}=0.204\frac{LET}{d^{2}}\left(\frac{keV}{\mu m}\right)$$ (10)

The nuclear diameter *d*, approximately 8 μm [50], was a typical size observed in V79 Chinese hamster cells used in various experiments [37]. LET is linear energy transfer.

**Fig 1:**
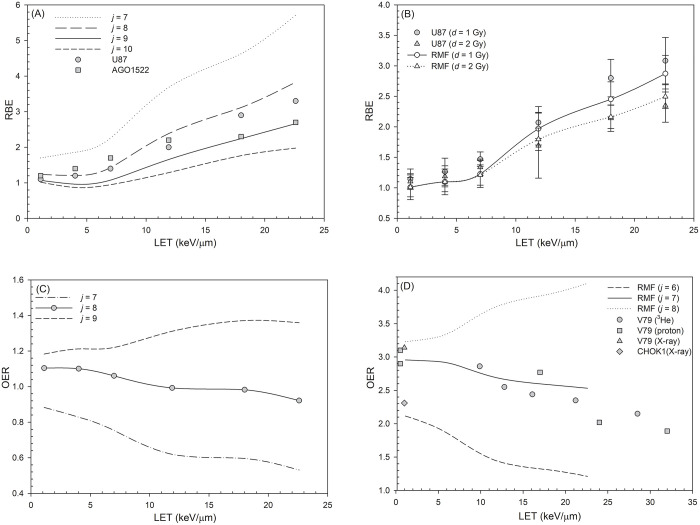
RBE values and OER values as a function of LET. **(A)** RBE values as a function of LET for RMF-model predictions using parameter *j* = 7–10. **(B)** RBE values as a function of LET for RMF-model predictions and *in vitro* data (U87 cell line [15]) for a single proton dose of 1 and 2 Gy for oxygen concentration 21%. **(C)** OER values as a function of LET for RMF-model predictions using parameter *j* = 7–9. **(D)** OER values at 10% cell survival were evaluated as a function of LET under 0.1% oxygen conditions. Experimental data were collected from previously studies involving X-rays, protons, and ^3^He ions for the V79 and CHO-K1 cell lines [43–46].

For this study, the parameter *j* has a value of 9 for 21% O_2_, is demonstrated in Fig 1(A) and 1(B). The parameter *j* was determined using a least-squares fitting procedure, in which the RBE values calculated from the Eq. (6) were compared with experimental data obtained from U87 and AGO1522 cells [15] for a LET value of 1–23 keV/μm. All calculations for the $\alpha_{p}$ and $\beta_{p}$ at a normal oxygen concentration (21% O_2_) or in hypoxic conditions (2% O_2_) use the values $\theta =5.79\times 10^{-3}$ and $\kappa =5.59\times 10^{-5}$, which were derived by a previous study [25].

#### OER calculations.

The OER for cell survival is defined in Eq. (11), where the doses, *D*_*a*_ (at an aerobic condition, 21% O_2_) and *D*_*h*_ (at a severely hypoxic condition (0.1% O_2_)) are calculated using Eqs. (12) and (13) [51], respectively, as shown below:


$$OER=\frac{D_{h}}{D_{a}}$$ (11)

Da=12{−(αaβa)+(αaβa)2−4(αa/αaβa\nulldelimiterspaceβa)ln(S)αa} (12)

Dh=12{−(αhβh)+(αhβh)2−4(αh/αhβh\nulldelimiterspaceβh)ln(S)αh} (13)

where $\alpha_{a}$, $\beta_{a}$, $\alpha_{h}$, and $\beta_{h}$ denote the LQ parameters under aerobic (21% O₂) and severely hypoxic (0.1% O₂) conditions, respectively, as defined in Eq. (2). Under the 2% O₂ condition, the parameter *j* was assigned a value of 8, as shown in Fig 1(C). All calculations for the $\alpha_{p}$ and $\beta_{p}$ at a hypoxic condition (2% O_2_) also use the values $\theta =5.79\times 10^{-3}$ and $\kappa =5.59\times 10^{-5}$, which were derived by a previous study [25].

The parameter θ, κ and *j* values for the 0.1% O₂ condition were determined by a least-squares fitting procedure, in which the OER values calculated from the Eqs. (11)–(13) were fitted to the corresponding experimental data, as shown in Fig 1(D). When the oxygen concentration decreases to 0.1%, the parameter *j* has a value of 7. For extremely hypoxic or anoxic conditions (0.1% O_2_), the calculations use the values $\theta =4.1\times 10^{-3}$ and $\kappa =3\times 10^{-5}$ [51].

#### EQD calculations.

Here, we define the dose modifying factor (DMF) as follows:


$$\text{DMF}=\frac{\alpha_{p}}{\alpha }$$ (14)

The EQD with fractionated dose [18] is expressed as:


$${EQD}_{DMF}=n_{prot}d_{prot}\frac{\left(DMF+\frac{d_{prot}}{\frac{\alpha }{\beta }}\right)}{\left(\text{1}+\frac{d_{low}}{\frac{\alpha }{\beta }}\right)}$$ (15)

where *d*_*low*_ (usually 2 Gy) is the dose per fraction for the reference photon schedule. The LET-independent β is assumed in Eq. (15). The rationale is due to that experimental evidence shows β varies only weakly with LET for low-LET protons (< 20 keV/μm) [5,14,20,52,53].

If the values for $\alpha_{p}$ and $\beta_{p}$ are calculated using a DSB-based RMF model [25], then the EQD can be expressed directly using the LQ parameters as:


$$EQD_{RMF}=n_{prot}d_{prot}\frac{\left(\alpha_{p}+\beta_{p}d_{prot}\right)}{\alpha \left(\text{1}+\frac{d_{low}}{\frac{\alpha }{\beta }}\right)}$$ (16)

The value for RBE for proton irradiations is assumed to have a generic value of 1.1, as recommended by International Commission on Radiation Units and Measurements [54] so the EQD [26] is calculated as:


$${EQD}_{clinical}=\text{1}.\text{1}n_{prot}d_{prot}\frac{\left(\text{1}+\frac{\text{1}.\text{1}d_{prot}}{\frac{\alpha }{\beta }}\right)}{\left(\text{1}+\frac{d_{low}}{\frac{\alpha }{\beta }}\right)}$$ (17)

The EQD formulations in Eqs. (15), (16), and (17) are based on distinct assumptions. Eq. (17) reflects the clinical assumption of constant RBE = 1.1 (EQD_clinical_), whereas Eq. (15) provides a phenomenological variable-RBE estimation through DMF (EQD_DMF_). Eq. (16), derived from the RMF model, is the mechanistically grounded formulation used for the main TCP results (EQD_RMF_).

#### TCP calculations.

The tumor control probability (TCP) [55] is calculated using the EQD and the dose response parameter $D_{50}$ and γ (gamma) as:


$$TCP=\frac{\text{1}}{1+{\left(\frac{D_{50}}{EQD}\right)}^{4r}}$$ (18)

where $D_{50}$ is the dose at 50% response probability. For the results in Tables 1–3, the number of proton treatments $n_{prot}=37$. The single dose *d*_*pron*_ for the proton treatment is 1.809 Gy. The respective values for $D_{50}$ and γ for the prostate are 59 ± 2.0 Gy and 1.04 ± 0.17 [55] for cells under aerobic conditions (21% O_2_). *D*_*50*_ are respectively set as 70.0 ± 2.6 Gy (using OER = 1.187 ± 0.004 [56]) and 177 ± 13 Gy (using OER = 3.0 ± 0.2 [57]) for cells under hypoxic conditions of 2% and 0.1% O_2_. The scaling of D₅₀ by fixed OER values represents a simplified approximation which allows comparison with existing prostate D₅₀ datasets, which are available only under aerobic conditions.

## Results

### RBE comparisons

Fig 1(A) and 1(B) show the RBE value versus LET. In Fig 1(A), the RBE calculated using *j* = 9 shows the closest agreement with the experimental data (see S1 Table for details). In Fig 1(B), the error-bar plot indicates that the RBE values obtained with *j* = 9 for proton doses of 1 Gy and 2 Gy are comparable to the experimentally measured RBE values. The RBE increases as LET increases, as shown in the *in vitro* U87 cell data [15] and the RMF-model predictions. For a single proton dose = 1 Gy, the RBE value for U87 cells increases from 1.1 ± 0.2 to 3.1 ± 0.4 as LET increases from 1 to 23 keV/μm, which is consistent with other findings [58–61]. For a higher single dose = 2 Gy, the RBE value for U87 cells increases to a smaller value of 2.3 ± 0.3 at LET = 23 keV/μm. As with U87 cells, the RMF model also shows a dependence on LET where 2.9 and 2.5 are the maximum values. These data also support the observation that a higher single dose results in a smaller RBE value [16,26]. In Fig 1(C), because experimental data on cell killing under hypoxic conditions are limited, a value of *j* = 8 was selected for cells irradiated at 2% O_2_ based on the observed trend that OER typically lies in the range of 1–3 and decreases with increasing LET. Fig 1(D) further demonstrates that the OER calculated using *j* = 7 provides the closest agreement with the experimental data.

### EQD and TCP comparisons

The effect of RBE on treatment outcomes in terms of EQD and TCP is also determined. Table 1 shows the results for the LQ parameter $\alpha_{p}$, $\beta_{p}$, EQD and TCP under aerobic conditions (21% O_2_). The values for the LQ parameter $\alpha_{p}$, $\beta_{p}$ increase as LET increases and the value of αp/αpβp\nulldelimiterspaceβp ratio is ~ 5 for LET = 1**–**7 keV/μm, but increases to 9 for higher-LET radiations. EQD_RMF_ ranges from 66 ± 13–233 ± 36 Gy and EQD_DMF_ from 65 ± 15–211 ± 52 Gy respectively, and differ by about 2**–**9%. EQD and TCP (as high as 98%) values also increase as LET increases to 12 keV/μm. Compared with a recent study [62], which showed an EQD of 71–97 Gy can achieve a TCP of 90–95%, our calculations are consistent with these findings. EQD_clinical_ is 74 ± 9 Gy and TCP is 71 ± 11%, which does not depend on LET or oxygen concentration.

Table 2 lists the values for $\alpha_{p}$, $\beta_{p}$, EQD and TCP under moderately hypoxic conditions (2% O_2_). The ratio of the two LQ parameters, αp/αpβp\nulldelimiterspaceβp, increases from 6.5 to 14.8 Gy as LET increases from 1.1 to 23 keV/μm. These values are greater than the ratio αp/αpβp\nulldelimiterspaceβp that are shown in Table 1 (21% O_2_). EQD_RMF_ ranges from 67 ± 15–299 ± 48 Gy and EQD_DMF_ from 66 ± 15–280 ± 56 Gy, respectively. EQD_RMF_ and its corresponding TCP value are greater than EQD_DMF_ and its TCP value. EQD_clinical_ is independent of LET and oxygen concentration and is constant at 74 Gy.

Table 3 lists the values for EQD and TCP under extremely hypoxic conditions (0.1% O_2_). As LET increases from 1 to 23 keV/μm, the ratio of the two LQ parameters αp/αpβp\nulldelimiterspaceβp rises from 16.9 to 34.6 Gy, exceeding the corresponding value shown in Table 2 (2% O_2_). EQD_RMF_ ranges from 64 ± 30–302 ± 102 Gy and EQD_DMF_ from 64 ± 30–293 ± 108 Gy, respectively. The respective TCP value intervals calculated using EQD_RMF_ and EQD_DMF_ are 1–90% and 1–89%, respectively.

**Fig 2:**
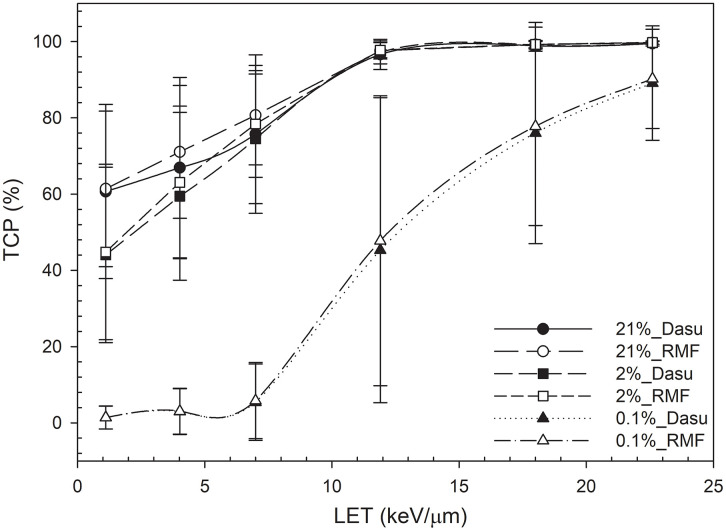
Tumor control probability (TCP) as a function of linear energy transfer (LET) for cells that are irradiated at oxygen concentrations of 21%, 2% and 0.1%.

Fig 2 shows the data from Tables 1–3, which list the TCP values at oxygen concentrations of 21%, 2% and 0.1%. For low-LET radiations, such as high energy therapeutic proton beams (e.g., 62 MeV, LET = 1 keV/μm) [15,63], the value of TCP using EQD_RMF_ for cells under aerobic conditions (21% O_2_) is highest (61 ± 20%) and decreases to 45 ± 23% for moderately hypoxic conditions (2% O_2_), and further to 1 ± 3% for cells under extremely hypoxic conditions (0.1% O_2_). For higher-LET radiations (e.g., lower energy protons, LET = 4 keV/μm), the value of TCP using EQD_RMF_ increases to 63 ± 20% (21% O_2_) and 3 ± 6% (0.1 O_2_). The TCP value decreases as the oxygen concentration decreases for low-LET radiations; however, as LET increases to 18 keV/μm, TCP increases to 78**–**99%, which demonstrates high-LET radiations are relatively insensitive to oxygen concentration.

The TCP values that are calculated using EQD_RMF_, EQD_DMF_ and EQD_clinical_ are compared with those reported from a major clinical study listed in Tables 4 and 5. Zietman *et al.* (2005, 2010) [47,48] studied 393 patients who were treated with a combination of photon and proton treatment that contained 28 photon fractions of 1.8 Gy and 11 (Table 4) or 16 (Table 5) proton fractions of 1.82 Gy. The clinical biochemical TCPs reported for two different regimens are 61.4% (11 fractions, 70.2 Gray equivalents (GyE); 70.2 Gy; conventional dose) and 80.4% (16 fractions, 79.2 GyE; high dose) [47]. A follow-up study [48] revealed that the 10-year biochemical TCPs were 78.4% for the conventional dose arm and 83.4% for the high-dose arm. In our work, the predicted TCP using EQD_RMF_ for 11 fractions (LET = 1 keV/μm) under hypoxic conditions (oxygen concentration decreasing from 21% to 0.1%) are 65–2%. For 16 fractions under the same conditions, the TCP increases to 74–3%. The clinical data provide qualitative consistency checks for our findings.

## Discussion

This study determines the effect of LET and oxygen concentration on TCP. A previous study by Dasu and Toma-Dasu (2013) [26] showed that the TCP values that are predicted using the LQ model are in good agreement with the TCP values for the clinical trial by Zietman *et al.* [47,48]. This study uses the LQ model with the parameters of the RMF model to predict the LQ model parameters α and β and shows that the TCP values depend on the LET and oxygen concentration. The following sections compare the results with other clinical trials and discuss the effects of LET, oxygen concentration and α/αβ\nulldelimiterspaceβ on TCP. The limitation of this study is also included.

### The effect of LET and oxygen concentration on TCP

Our results, summarized in Tables 1–3, demonstrate how increasing LET impacts TCP under different oxygen conditions. These findings align with previous studies, including Garrido-Hernandez *et al.* (2022) [64], which showed that OER decreases as LET increases, and the effect of hypoxia diminishes for high-LET radiations. However, the RMF model relies on several parameters that are known to be sensitive, including RMF parameters (θ, κ, j), LQ parameters (α, β), oxygen-dependent corrections. The variations in these parameters can substantially influence RBE, EQD, and ultimately TCP predictions. In Table 1, the resulting variations in TCP for low-LET protons (1 keV/μm) is less than 20% but become larger under extreme hypoxia, highlighting the model’s sensitivity to hypoxia-related parameters.

Importantly, our analysis using the RMF model reveals that hypoxia has a substantial effect on TCP for low-LET radiations, supporting earlier observations [36,46]. The TCP value is less affected by oxygen concentration for high-LET radiations because the OER values for cells that are irradiated by high-LET radiations are smaller. The OER value is ~ 3 for low-LET radiations and decreases to 1–2 for high-LET radiations so it decreases as LET increases. The OER value is higher for low-LET radiations because of its involvement in indirect actions [7,65]. Two third of the yield for DNA damage that is induced by low-LET radiations is due to indirect actions [11], in which oxygen plays a key role through the formation of free radicals and the repair of DNA damage [33,66,67]. However, in the work of Garrido-Hernandez *et al.* (2022) [64], it was shown that OER decreases as a function of LET but there was only a weak LET dependence of the OER in the low LET range (1–11 keV/μm). Consistently, the data by Furusawa *et al.* [45] show that the OER is pretty much constant for protons in the LET range of 1–18 keV/μm. The relationship between OER and LET seems neither universally monotonic nor simple; under certain conditions, it may be weak, ion-species dependent, or vary with the choice of endpoint and model system [68]. Some *in vitro* and *in vivo* studies report persistent oxygen sensitivity or only modest reductions in OER with increasing LET, highlighting the complexity of OER behavior across different experimental settings [43].

By contrast, for proton beams, the RBE value for proton beams increases as oxygen concentration decreases [56] because the reduction in DSB yields, or equivalently in radiation dose, is more pronounced for the reference radiation such as ^60^Co. In this case, the EQD, which is the RBE-weighted dose, behaves similarly to RBE, and increases as the oxygen concentration decreases. The TCP decreases as the EQD increases and the overall TCP decreases as the oxygen concentration decreases [36,69].

It has been shown that the EQD_DMF_ gives a greater RBE for hyper-fractionated schedules (lower single dose) [26,70]. However, the clinical outcomes for recent studies show no significant advantages for hyper-fractionated schedules over conventional radiation therapy (RT) [71–73]. The medical outcome for the hyper-fractionated accelerated RT features a high overall survival rate but the result is not statistically significant. Low-LET PT using 150–210 MeV proton beams shows results comparable to conventional RT [74], possibly due to the radioresistant nature of hypoxic tumors, which exhibit a high α/β ratio and a low TCP. In addition, Wang *et al*. [75] also showed that the TCP decreases from ~ 65% to 2% as the hypoxia fraction increases from 10% to 50%. The TCP decreases as the initial tumor volume increases for head and neck cancer [76], non-small cell lung cancer [42], melanoma [77] and cervical cancer [78]. Further analysis shows that the volume only affects hypoxic tumors and has a negligible effect on oxygenated tumors [34]. Tumor hypoxia heterogeneity and cycling hypoxia play critical roles in modulating TCP. Spatial hypoxia heterogeneity generally reduces the α parameter in the LQ equation, leading to a downward shift of the TCP curve, whereas cycling hypoxia induces larger temporal fluctuations in OER [40,42,66], thereby further decreasing TCP.

One commonly proposed approach to mitigate tumor hypoxia is dose escalation to hypoxic subvolumes, a strategy referred to as dose painting [37,75,76,79,80]. In the context of radiotherapy for hypoxic tumors, redistributing the LET to reduce oxygen dependence—so-called LET painting—has also been suggested [81–83]. LET painting conceptually aims to preferentially deliver higher-LET radiation to hypoxic regions while limiting LET in normoxic tissues, thereby potentially enhancing biological effectiveness without increasing the total physical dose. However, LET painting remains largely investigational, as its clinical realization is constrained by uncertainties in hypoxia imaging, LET prediction, patient motion, and the modeling of LET-dependent biological effects.

### The effect of $\frac{\alpha }{\beta }$ ratio on RBE and TCP

In Eqs. (8) and (9), the α/αβ\nulldelimiterspaceβ ratio for cells that are irradiated with X-rays is used to calculate the EQD. The α/αβ\nulldelimiterspaceβ ratio is used to determine sensitivity to radiations. α/αβ\nulldelimiterspaceβ=10 Gy give an appropriate sensitivity for fast-growing tumors or an acute reaction for normal tissues and α/αβ\nulldelimiterspaceβ = 3 Gy is appropriate for slow-growing tumors and late-reacting normal tissue. The study by Dasu and Toma-Dasu [26] showed that the RBE increases from 1.5 to 2.2 as LET increases from 3 to 10 keV/μm for cells with α/αβ\nulldelimiterspaceβ = 1.5 Gy while the RBE increases from 1.1 to 1.4 for cells with α/αβ\nulldelimiterspaceβ = 10 Gy. This shows that higher RBE values than 1.1 are particularly affected for cells with a low α/αβ\nulldelimiterspaceβ ratio, such as slowly-growing tumors and late-reacting normal tissue but only slightly affect acutely-reacting tissue. Motivated by these findings, we performed a sensitivity analysis to evaluate whether variations in the assumed X-ray α/β values propagate into clinically relevant EQD and TCP estimates. Using Eqs. (17) and (18), we found that the clinical EQD and corresponding TCP values remain remarkably stable across a plausible range of X-ray α/β values (Table 6). Importantly, the resulting trends are consistent with those observed in Tables 1–3, indicating that the qualitative conclusions of the present study are robust with respect to uncertainties in the assumed α/β ratio.

**Table 6:** Sensitivity analysis of EQD_clinical_ and TCP for X-ray α/β = 3 and 10 Gy under different oxygen levels (0.1–21% O₂).

Oxygen concentration (%)	X-ray $\frac{\alpha }{\beta }$ (Gy)	EQD_clinical (Gy)	TCP(%)
21	3	73	71
2	3	73	55
0.1	3	73	3
			
21	10	74	71
2	10	74	55
0.1	10	74	3

The TCP values that are calculated using EQD_RMF_, EQD_DMF_ differ slightly (up to 9%). The LQ parameters α and β are often respectively treated as one-track and two-track cell killing mechanisms. For low-dose, low-dose rate and hypoxic conditions, the role of the two-track cell killing mechanism is insignificant [52,84,85]. In the RMF model, the β term also arises primarily from two-track processes, which are therefore suppressed under hypoxia and at clinically relevant low doses per fraction. Consequently, low-LET proton therapy—particularly at conventional fractionation (~1.8 Gy per fraction)—is dominated by one-track (α-type) killing. Under these conditions, and especially in the presence of hypoxia, the influence of the β component becomes minimal, leading to similar TCP predictions from the EQD_RMF_ and EQD_DMF_ formulations. This behavior is consistent with previous theoretical analyses and simulation studies of DNA damage and repair [25,52,84,85].

Similarly, Wedenberg et al. (2013) [14] primarily modeled LET dependence through the α parameter rather than β. Paganetti (2014) [5] also reported that, for clinically relevant proton beams, β exhibits substantially weaker LET dependence compared with α. Nevertheless, several radiobiological datasets (e.g., V79, CHO, and TK1 cell lines) have demonstrated measurable increases in β with increasing LET, even within the low-LET region (< 20 keV/μm) [86]. To address this potential concern, we performed a sensitivity analysis by assuming a conservative 30% increase in β for protons relative to X-rays (i.e., β_proton = 1.3 × β_X-ray), corresponding to the upper bound reported by Friedrich et al. (2013) [86] for LET < 20 keV/μm. Under this assumption, the resulting change in the calculated TCP values (using EQD_RMF_, EQD_DMF_ and EQD_clinical_) was less than 7%, indicating that moderate variations in β have a limited impact on TCP in the present model. In contrast, variations in α produced substantially larger changes in TCP, consistent with previous findings [5,26]. We note that β may increase more significantly at higher LET values, which could lead to a stronger influence on TCP. However, such high-LET effects are beyond the scope of the current study, which focuses on clinically relevant low-LET proton beams.

### The limitation

Our method provides a flexible tool for calculating TCP across a range of oxygen concentrations and LET values. However, scaling D₅₀ using a fixed OER constitutes a simplified approximation that may overestimate hypoxia-induced penalties at higher LET. Experimental evidence indicates that high-LET protons exhibit OER values close to 1–1.5, rather than 3, even under severe hypoxia (0.1% O₂). Consequently, applying a constant D₅₀/OER scaling should be regarded as an upper-bound estimate of hypoxia effects, and TCP predictions at higher LET should be interpreted conservatively.

In addition, the present model assumes uniform hypoxia, hence its future extensions of this model will need to incorporate heterogeneous oxygenation distributions, potentially through voxel-level hypoxia weighting functions or probabilistic cycling-hypoxia terms. Incorporating spatial hypoxia distributions (e.g., via voxelwise OER maps) requires patient imaging or microvascular geometry assumptions. Furthermore, direct clinical α/β values for the specific tumor type and treatment modality investigated in this study are currently not available. The mixture of experimental α/β values with clinical TCP parameters introduces biological uncertainty [26,27]. The RMF model is subject to additional sources of uncertainty, including the choice of RMF parameters (θ,κ,j), the LQ parameters (α,β), and oxygen-dependent correction factors. Variations in these inputs can propagate through the RMF–EQD–TCP framework and substantially affect RBE, EQD, and ultimately TCP predictions. As a result, the high-LET results presented in Tables 1–3 are intended to illustrate model behavior across the full physical LET domain and to support mechanistic interpretation, while the comparison with clinical prostate data (Tables 4 and 5) is confined to the low-LET regime relevant to clinical practice.

## Conclusion

We have developed a computational method that accounts for varying oxygen concentrations and LET values. Our results show that hypoxia significantly lowers TCP for low-LET radiations but has a negligible effect on higher LET radiations. These findings provide new insights into optimizing radiotherapy for hypoxic tumors.

## Supporting information

S1 File MCDS input and output files for oxygen concentrations of 0.1%, 2%, and 21%. These files include the DSB yields simulated by MCDS and the proton spectra used at different LET positions. Available at: https://nfuedu-my.sharepoint.com/personal/ccchan_nfu_edu_tw/_layouts/15/onedrive.aspx?id=%2Fpersonal%2Fccchan%5Fnfu%5Fedu%5Ftw%2FDocuments%2Fplos%20one&ga=1.
(ZIP)

S1 Table RBE values using a least-squares fitting procedure, in which the RBE values calculated from the Eq. (6) were compared with experimental data obtained from U87 and AGO1522 cells. (PDF)
