# Supplementary material for: A computational method to estimate the relative biological effectiveness and tumor control probability for low-LET proton irradiations
Source: PLoS One. 2026 Mar 20;21(3):e0341352. doi: 10.1371/journal.pone.0341352 (PMC13004361; doi:10.1371/journal.pone.0341352)
Supplement: S1 Table — (PDF) [file pone.0341352.s002.pdf]

S1 RBE values using a least-squares fitting procedure, in which the RBE values calculated from the Equation (6) were compared with experimental data obtained from U87 and AGO1522 cells [15]

LET (keV/μm)	RBE						least square	least square
	j=7	j=8	j=9	j=10	U87	AGO1522	j=8	j=9
1.11	1.70	1.24	1.07	1.02	1.1	1.2	0.0196	0.0175
4.02	1.86	1.21	0.96	0.87	1.2	1.4	0.0376	0.2482
7	2.25	1.43	1.08	0.95	1.4	1.7	0.0754	0.4942
11.9	3.68	2.38	1.66	1.29	2	2.2	0.1824	0.4151
18	4.63	3.12	2.24	1.76	2.9	2.3	0.7128	0.4361
22.6	5.71	3.83	2.66	1.98	3.3	2.7	1.5535	0.4051
							sum	sum
							2.5814	2.0162
